# Supplementary material for: Ensemble deep learning of embeddings for clustering multimodal single-cell omics data
Source: Bioinformatics. 2023 Jun 14;39(6):btad382. doi: 10.1093/bioinformatics/btad382 (PMC10287920; doi:10.1093/bioinformatics/btad382)
Supplement: btad382_Supplementary_Data [file btad382_supplementary_data.pdf]

Supplementary Figures

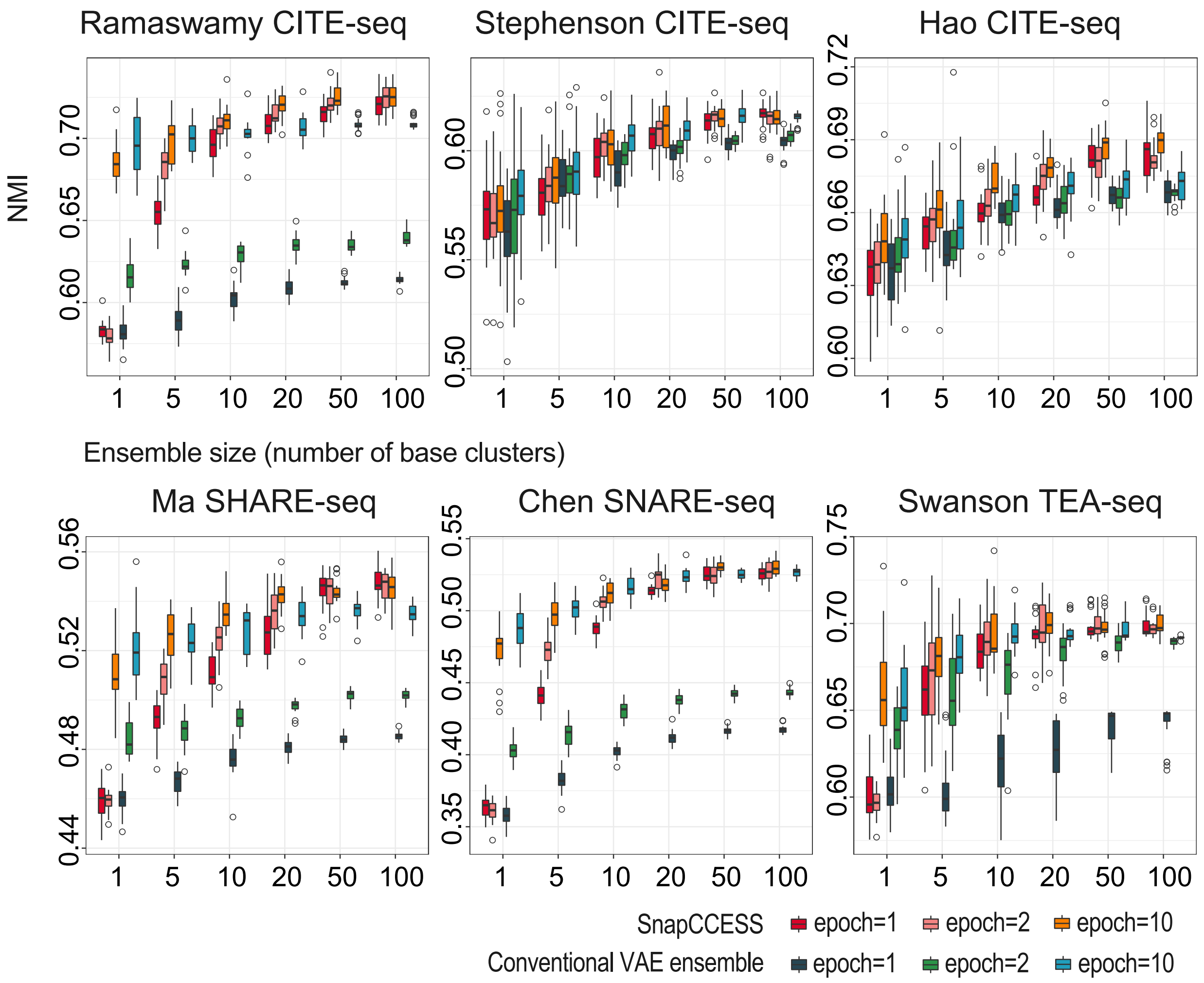

Fig. S1: Concordance of cell type clustering on six multimodal single-cell omics data. The x-axis is the number of base clusters for the ensemble and the y-axis is the concordance of the clustering output and the cell type annotation in the original studies quantified by NMI. The k-means clustering algorithm was used for clustering the embeddings generated from each method. The entire procedure was repeated 20 times for capturing the performance variability.

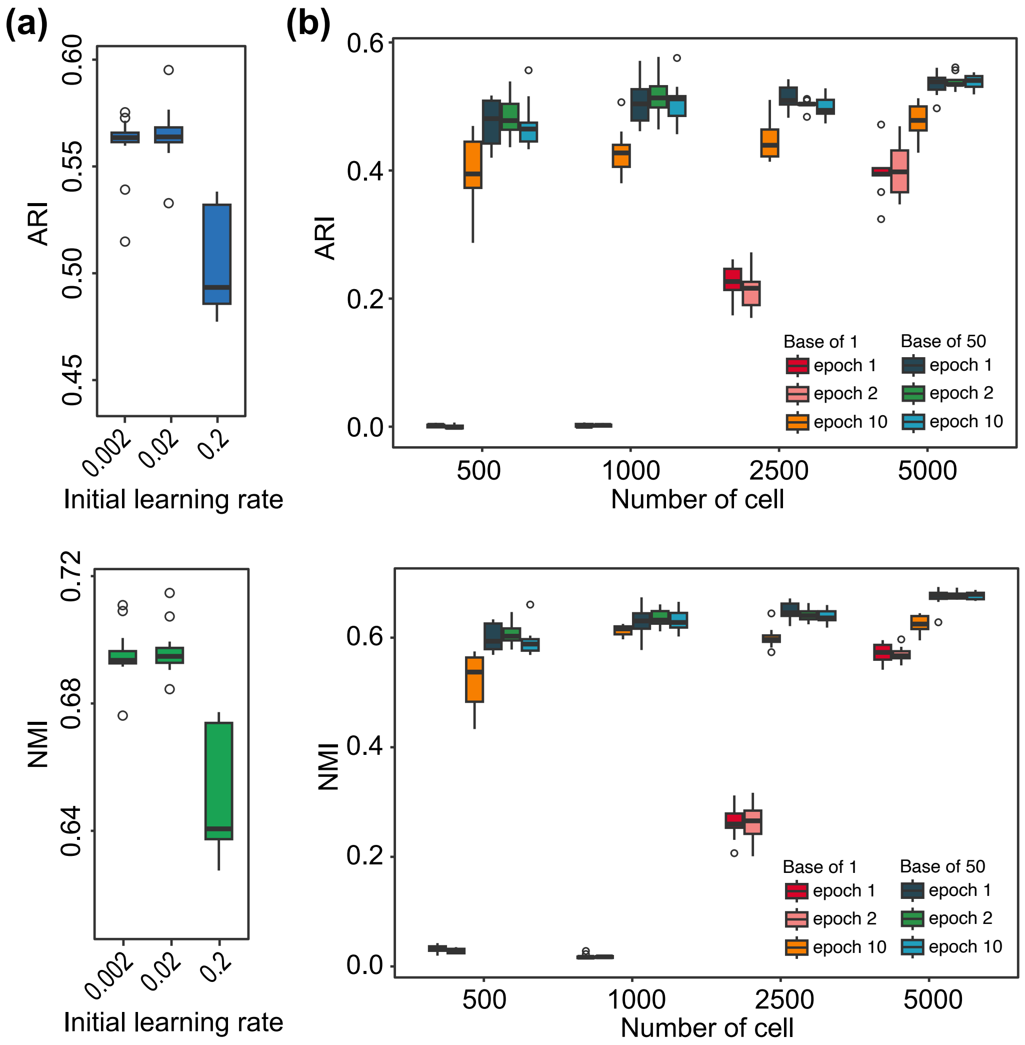

Fig. S2: Evaluation of performance on (**a**) varying initial learning rates, and (**b**) the numbers of cells using the TEA-seq dataset by Swanson et al. Concordance of clustering output and the pre-defined cell type labels were quantified using ARI and NMI.

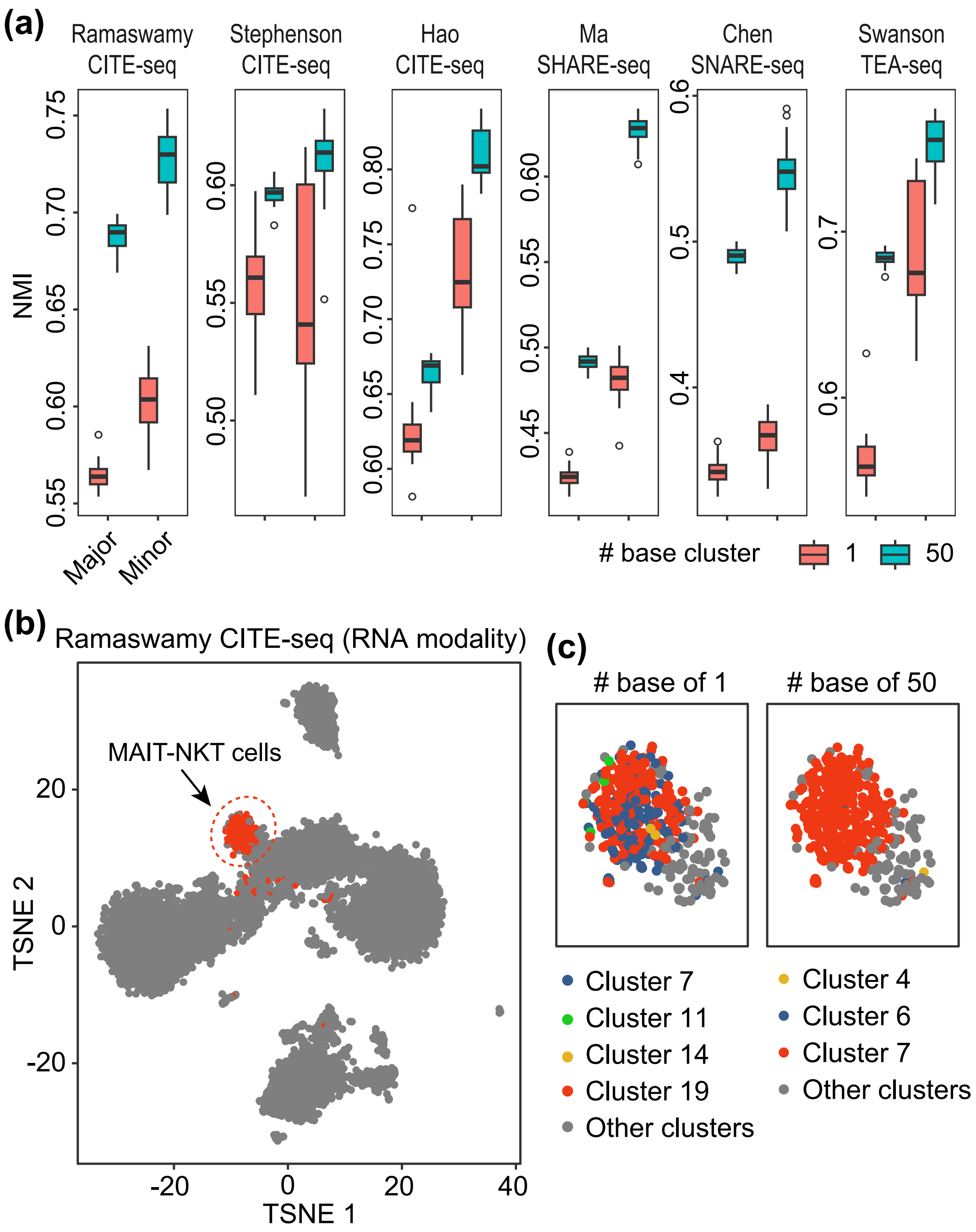

Fig. S3: Evaluation of SnapCCESS (epoch=1) performance on (**a**) major and minor cell type clustering using each of the six datasets. Concordance of clustering output and the pre-defined cell type labels were quantified using NMI. (**b**) TSNE visualisation of Ramaswamy CITE-seq data using RNA modality. MAIT-NKT cells are highlighted in red. (**c**) Zoom in of MAIT-NKT cell clustering using SnapCCESS with 1 or 50 base clusters.

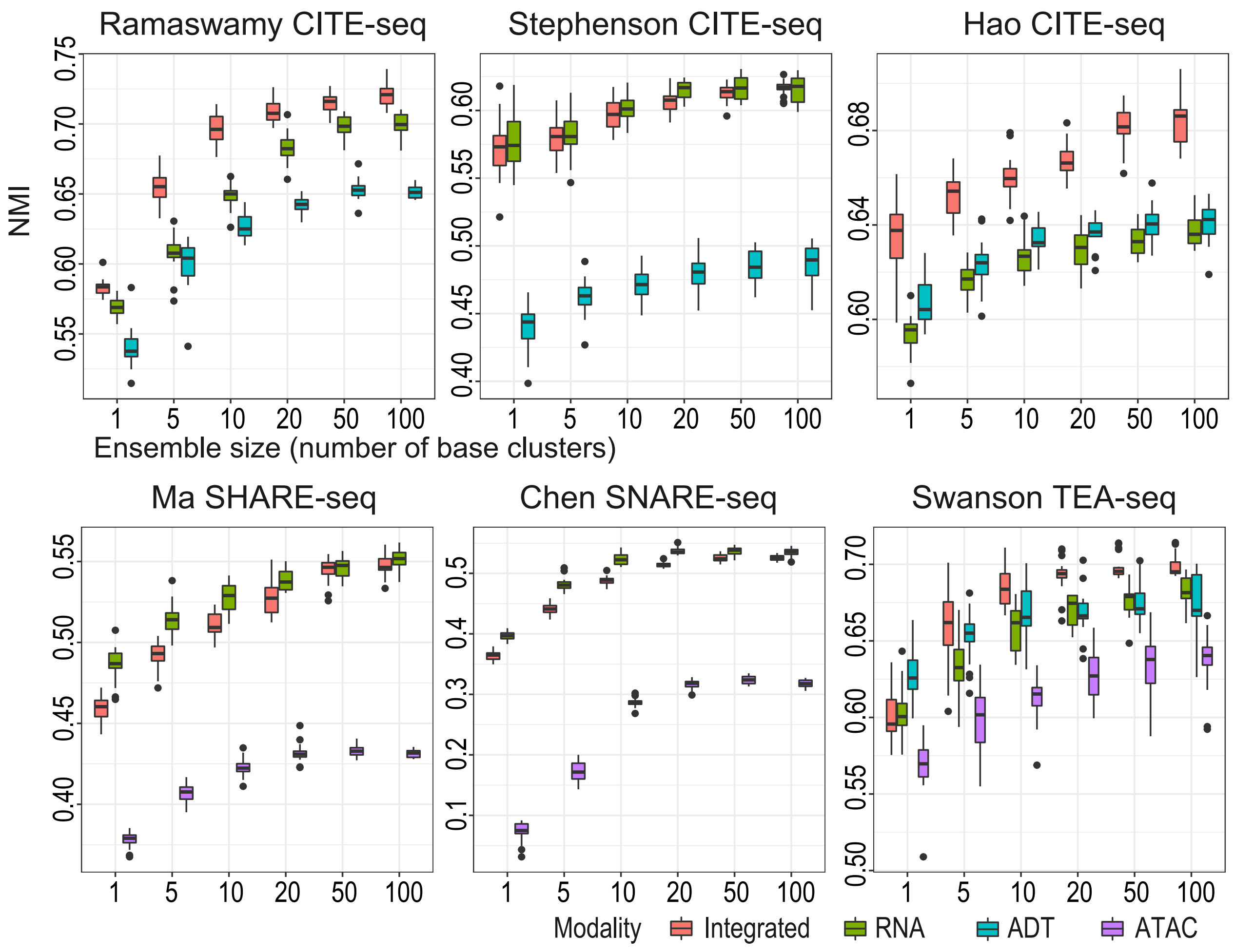

Fig. S4: Comparison of integrated embedding of multimodality learned with those from unimodality using the k-means clustering algorithms. Concordance of cell type clustering quantified by NMI on six multimodal single-cell omics data using SnapCCESS generated embeddings from either all modalities in a dataset or each data modality alone. The entire procedure was repeated 20 times for capturing the performance variability.

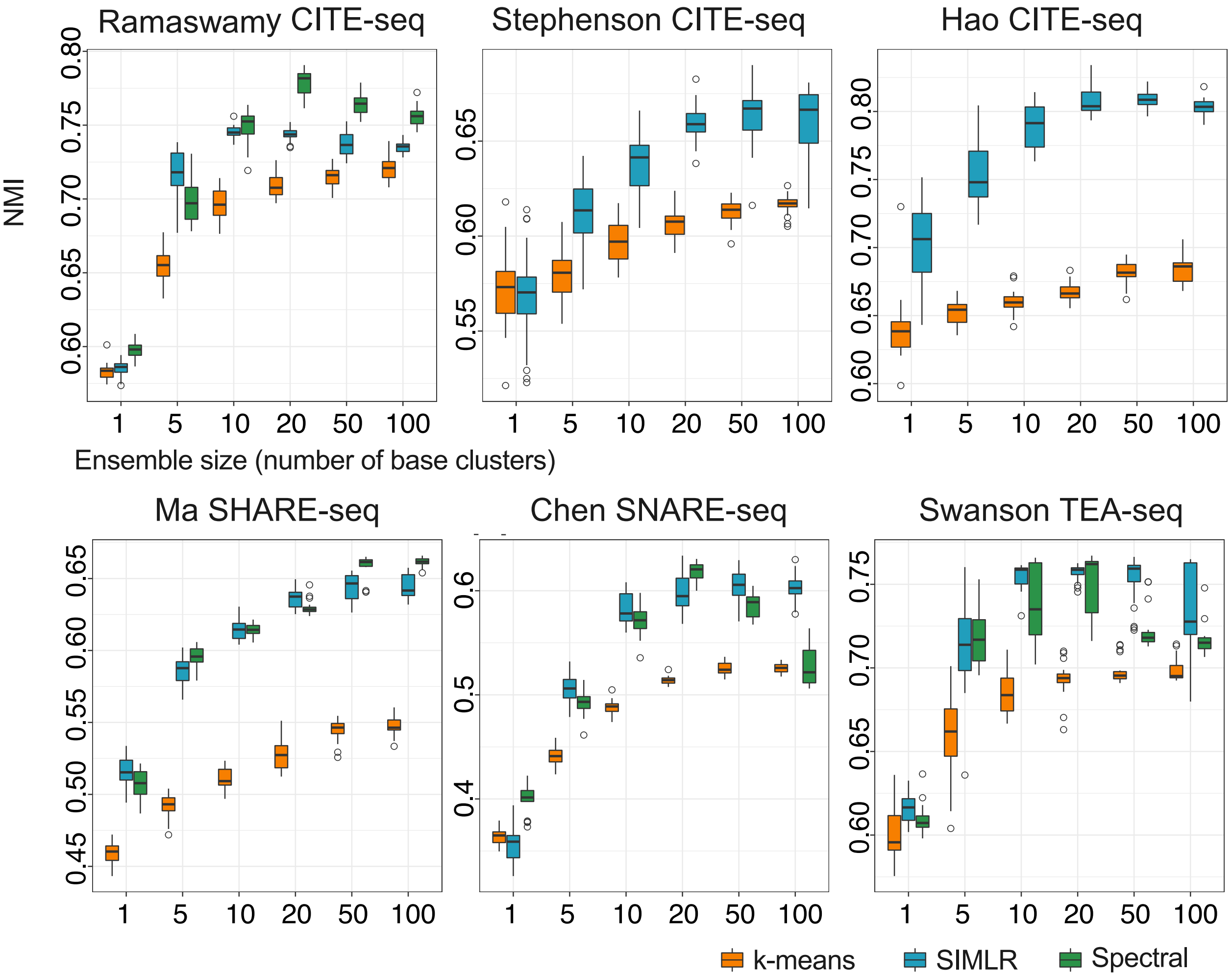

Fig. S5: Evaluating the utility of the SnapCCESS framework with alternative clustering algorithms on multimodal single-cell omics data. Concordance of the cell type annotations and cell clustering output from each clustering algorithm. The x-axis is the number of base clusters in the ensemble and the y-axis is the concordance quantification by NMI. This procedure was repeated 20 times for capturing the performance variability.
